# Thermal Evaluation of Scorched Graphite-Epoxy Panels by Infrared Scanning

**DOI:** 10.6028/jres.108.015

**Published:** 2003-04-01

**Authors:** A. J. Slifka, T. Hall, E. S. Boltz

**Affiliations:** National Institute of Standards and Technology, 325 Broadway, Boulder, CO 80305; TPL, Inc., Albuquerque, NM 87109

**Keywords:** composites, graphite-epoxy, infrared imaging, thermal performance

## Abstract

A simple measurement system is described for evaluating damage to graphite-epoxy panels, such as those used in high-performance aircraft. The system uses a heating laser and infrared imaging system to measure thermal performance. Thermal conductivity or diffusivity is a sensitive indicator of damage in materials, allowing this thermal measurement to show various degrees of damage in graphite-epoxy composites. Our measurements track well with heat-flux damage to graphite epoxy panels. This measurement system, including analysis software, could easily be used in the field, such as on the deck of an aircraft carrier or at remote air strips.

## 1. Introduction

The widespread acceptance of graphite-epoxy composite components in aerospace structures has resulted in considerable gains in performance over that of conventional materials. The understanding of the limitations of composite structures, however, is somewhat limited with understandable emphasis on mechanical performance of new and aged components. Although significant work has characterized the thermal performance of certain components, only recently have efforts focused on degradation of mechanical performance resulting from high thermal flux.

The motivation for these studies of heat-induced mechanical degradation is the concern about dramatically compromised structural integrity accompanied by no obvious visual indicators. Although there are some obvious situations, such as carrier-deck fuel fires, that require detailed investigation of the exposed structure, there are less obvious situations for concern. For example, it is not uncommon for the crowded deck of an aircraft carrier to produce a situation where the exhaust of one aircraft is directed onto and in close proximity to a composite structure on an adjacent aircraft. Debonding of fibers and matrix, matrix degradation, and delamination cause a loss of strength in composite materials under this type of demanding application.

A relatively simple and preferably nondestructive method is needed to evaluate the degree of damage to the material. Measurement schemes can be found in the literature that have varying degrees of complexity and yield results that are more or less quantitative [1, 2]. Recent work has been done in thermographic nondestructive evaluation (NDE) methods for many classes of materials, including composite structures [3–6]. Since damage results in local thermal barriers, we decided to use a quantitative measurement of relative heat transfer in these panels to show a correlation between damage and thermal conduction. The measurement method presented here is also amenable to making the measurements on the deck of an aircraft carrier or other similar environment and the subsequent analysis could easily be carried out in the field.

## 2. Experimental Method

We used an infrared (IR) scanning system to observe the transient thermal behavior of 12 (3 sets of 4 each) graphite-epoxy panels of which a third were obtained from the Air Force and the rest obtained commercially. The panels were 10 cm by 10 cm square, ranging from 2.38 mm to 4.76 mm thick. One thin specimen set, designated “A”, were unidirectional laminates, while the other thin specimens, designated set “B”, and the thick specimens, designated set “C” were quasi-isotropic laminates where there were an equal number of fibers at 0°, 90°, +45°, and −45°. The thin material was AS4/3501-6, and the thick material was IM7/3501-6. The thin material had a fiber volume fraction of 0.63 and that for the thick material was 0.65. Each set of samples came from the same parent plate and were machined to minimize edge damage.

**Fig. 1:**
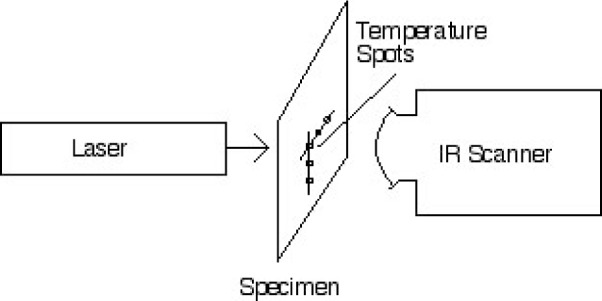
Schematic drawing of the experimental setup showing the temperature spots, arranged orthogonally, which are viewed by the scanner.

The thick panels had been exposed to a jet-fuel burner for decreasing lengths of time, whereas the thin specimens were baked for decreasing times using quartz-filament heaters, ending with one undamaged specimen in each set. Our measurement consisted of heating the backside of each specimen with a carbon dioxide laser and observing the temperature response at the front side. We used an 8.5 W pulse of 3 s duration for the thin specimens and a 5 s, 12 W pulse for the thick specimens. This amount of heat over this short amount of time will not cause appreciable damage to the specimens. We recorded the temperature response of the center of the spot opposite the point where the laser illuminated the specimen, and in many cases, collected temperature data at points 13 mm and 26 mm from the center of illumination. Figure 1 shows a schematic drawing of the simple experimental setup used to make these measurements.

## 3. Data Analysis

**Figure 2:**
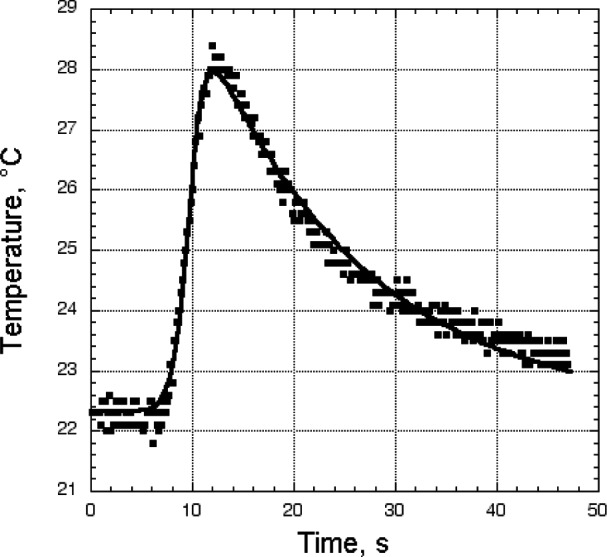
Raw temperature data from the IR scanner and the best curve-fit of the data for a thin, quasi-isotropic specimen.

We plot the temperature-versus-time data using commercial software to fit the temperature-pulse data. This particular software fits the data to over 3400 different functions, from which we can choose the most desirable fit. Many commercial software systems are capable of doing curve-fitting that is adequate for this application. Figure 2 is a plot of data from the scanner showing typical data scatter due to thermal noise. Each specimen set was fitted to a single functional form for a valid comparison. In each case, the functional form used to fit the data was either the best or second-best fit for all four tests (specimens) in a set, and usually the best fit for all four tests.

The IR system records temperature data at a rate of six measurements per second. Each temperature transient represents between 250 and 280 data points. No data averaging was used because the method should be fast and easy to use. A few panels were measured eight separate times, which resulted in better than 1 % repeatability for the measurements.

**Fig. 3:**
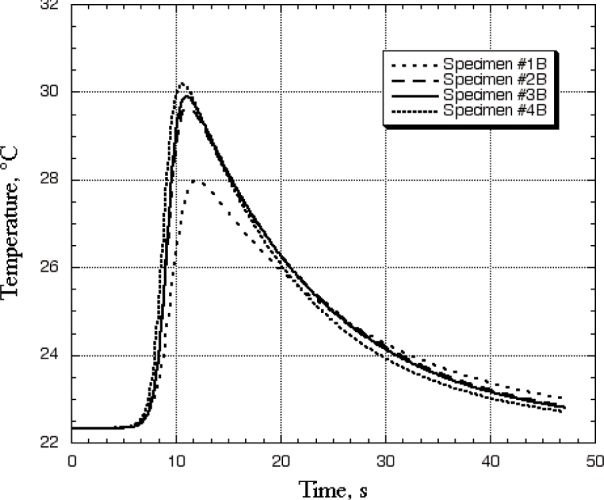
Fitted curves showing temperature profiles for the set of thin, quasi-isotropic specimens.

One could analyze the fitted curves in a number of different ways. One way was to look at how the curves for temperature as a function of time drop after the temperature peak. A damaged specimen should have a lower thermal conductivity than an undamaged or less damaged one, so for specimens with more damage the temperature should drop off more slowly after the temperature peak. This damage would be in the form of matrix cracks, fiber disbonds, or delaminations. At long times, the data for a set of four specimens would show up on a plot ranked from lowest to highest conductivity inversely relative to the temperature axis because the efficiency of heat loss is what is measured at long times. Therefore a panel of high thermal conductivity would show a higher temperature spike as the heat flowed unimpeded to the detection point on the backside of the panel, then the temperature would fall rapidly; whereas a low-conductivity panel would show a smaller temperature-rise peak followed by a lower rate of temperature decrease due to low thermal conductivity. The conductivity of the composite specimen serves to promote the heat-loss mechanisms because the panel acts like a heat-exchange fin. The thermal property trend can be seen in Fig. 3, which shows the fitted curves corresponding to data from a thin, quasi-isotropic specimen. The time corresponding to the peak temperature will increase as the thermal conductivity decreases, a trend which can be seen in this same plot. Probably the most useful and easily observable datum is the peak temperature difference, which is the difference between the test starting temperature (room temperature) and the maximum peak value. A specimen with higher conductivity will show a larger peak temperature difference, and consequently, specimens with increasingly larger amounts of damage will show smaller peak temperature differences.

## 4. Results

Figure 3 showed that as the specimen number within the set of thin, quasi-isotropic specimens increases, there is probably a decreasing amount of scorching damage to the specimens. Specimen #1B had obvious damage to the naked eye, whereas the other three specimens visually appeared to have decreasing amounts of damage when compared to one another. However, if specimens #2B-4B were in place on an aircraft, scorching damage might not be discernable by visual inspection. For further analysis, we normalized the peak temperature differences using specimen #4B, which had no damage and had the largest peak temperature difference. Specimen #1B, which showed obvious heat damage, had a 28 % drop in peak temperature difference. Specimen #2B had a 7 % drop. The 4 % drop observed for specimen #3B may not be significant since the relative standard uncertainty of the temperature measurement is 4 %, based on the standard uncertainty of the fits for the four runs. The numeric data for spots 13 mm and 26 mm away from the laser-heated spot showed a similar trend with less signal due to the distance between the laser and recording spots. Since this set of specimens was quasi-isotropic, it is not surprising that the data recorded away from the laser-heated spot yielded no new information.

**Fig. 4:**
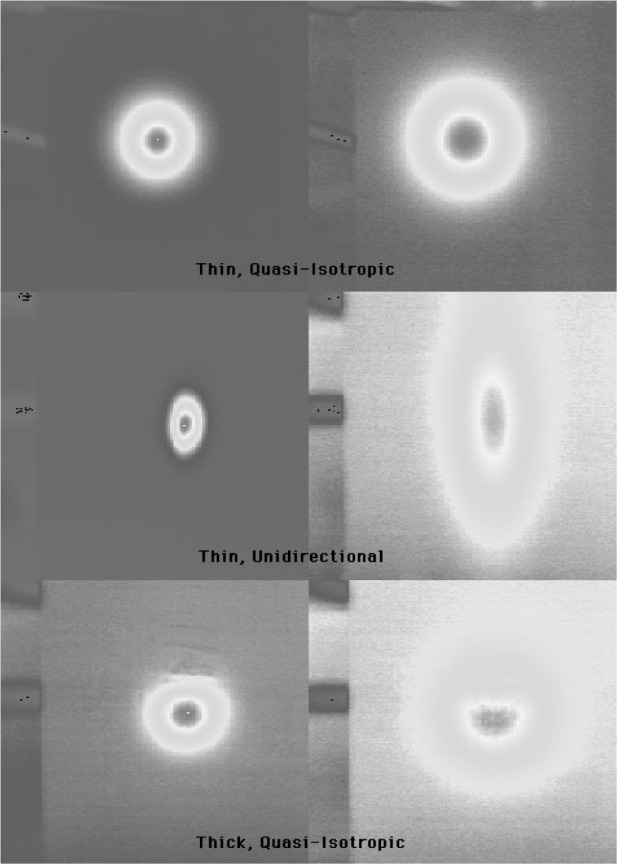
Infrared images of representative panels from each of the three sets of specimens.

**Fig. 5:**
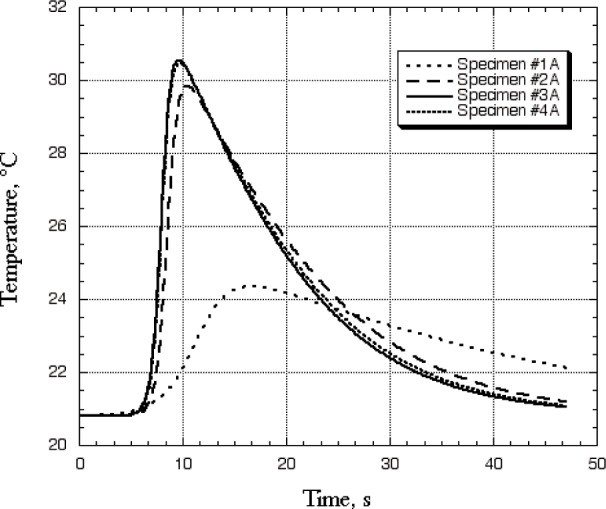
Fitted curves showing temperature profiles for the set of thin specimens with a unidirectional lamination.

Figure 4 shows infrared images with a representative panel from each of the three specimen sets. The images on the left were made with the specimen at peak temperature and the images on the right were taken well after the peak, when much of the heat had conducted and radiated away. These infrared images show that the fiber layup of the second set of thin specimens is non-isotropic. The fibers are unidirectional according to the manufacturer. Figure 5 shows the temperature profile curves for the set of thin, unidirectionally laminated specimens. These temperature profiles were recorded from the spot corresponding to the laser heating spot. The specimen that shows the most damage to the naked eye (#1A) has a fitted temperature profile curve that indicates a large amount of damage, as the peak time is significantly delayed and the peak temperature difference is over 63 % less than for the undamaged specimen in the set. Specimen #2A in the set of thin, unidirectionally laminated specimens shows a 6.5 % drop, which is statistically significant, given the 4 % relative standard uncertainty in the peak temperature-difference measurement. Specimens #3A and #4A in this set are indistinguishably close in peak temperature difference.

**Fig. 6:**
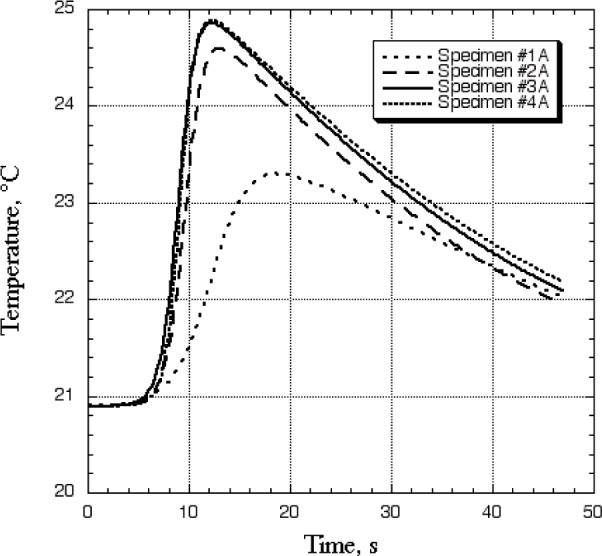
Fitted curves showing temperature profiles 13 mm away from the laser heating spot on a path in the direction of the fibers for the set of thin, unidirectionally laminated specimens.

Figure 6 shows temperature profile curves for the thin, unidirectionally laminated specimens for a spot 13 mm away from the laser heating spot, along the direction of the fibers, which is in the direction of high thermal conductivity. Specimen #1A is obviously damaged to the naked eye, and shows a peak temperature drop of 40 %. Specimen #2A shows a 7.4 % drop, and specimens #3A and #4A are statistically indistinguishable. These data are similar to data recorded at the spot corresponding to the laser heating spot because the fibers run in a direction from one recording spot to the other, and this is the preferred heat path. The functional form of the best-fit function is even the same for both temperature recording spots.

**Fig. 7:**
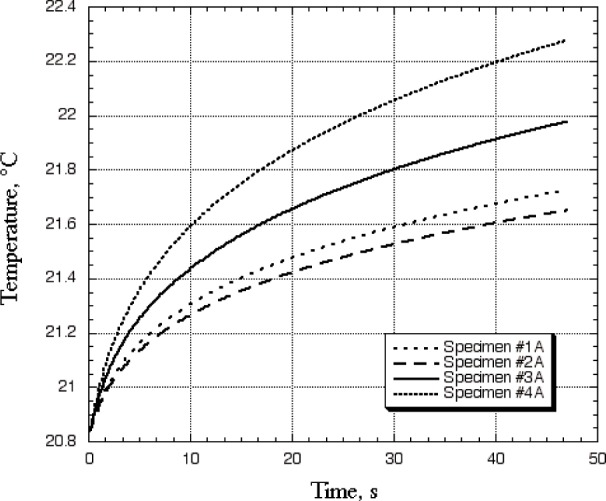
Fitted curves showing temperature profiles for a thin, unidirectionally laminated set of specimens 13 mm away from the laser heating spot on a line perpendicular to the fibers.

A plot of the fitted temperature profiles for a spot 13 mm away from the laser heating spot in a direction perpendicular to the fibers is shown in Fig. 7. For this type of curve shape, a higher thermal conductivity specimen would show a more rapid temperature rise, indicating less damage from the thermal exposure. The thermal conductivity in this direction is so much lower than for the fiber direction, there are no peaks even after 30 s, as the heat is preferentially transferred away in a different path. Two things should be noted from the figure, however. The thermal performance in this direction is significantly better for specimen #4A than for specimen #3A, which indicates that there is probably some damage to the epoxy matrix but not to the fibers in specimen #3A. A possible anomaly is seen in the data for specimen #2A, which shows a significantly lower temperature curve than specimen #1A. Even though specimen #2A has had less thermal exposure than specimen #1A, there is probably a large thermal defect, such as a transverse crack in the matrix between the laser spot and the detector spot, that gives this result. Figure 7 shows that different but meaningful results can occur at detector spots away from the laser heating spot.

**Fig. 8:**
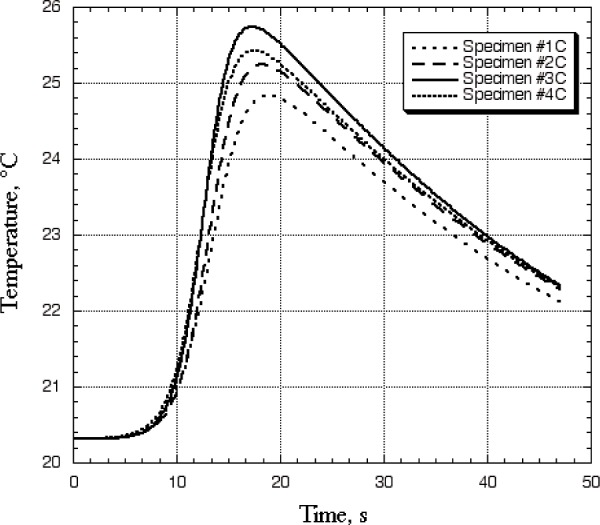
Fitted curves showing temperature profiles for the set of thick, quasi-isotropic specimens.

The thick, quasi-isotropic specimens were heated with the laser at full power, 12 W, for a 5 s duration. We limited the duration of the laser pulse to 5 s to ensure that no additional damage would be caused by the measurement. A higher-power laser used for a shorter duration would probably produce better results for the thick specimens. Figure 8 shows the resulting temperature profile data. Specimen #1C showed a nearly 12 % drop, compared to specimen #4C, which was the undamaged sample of the set. Specimen #2C showed a 3.7 % drop, which was on the edge of being statistically significant. Specimen #3C showed a 6 % rise in temperature peak difference, which was not expected. We made three additional measurements on this set of specimens, and the fitted temperature profiles agreed to within 0.5 %. It may have been that specimen #3C had a fairly short thermal exposure, so that high temperature did not result, or at least not for very long, so the effect was to cure the epoxy or increase the epoxy-fiber or fiber-fiber contact, raising the thermal conductivity slightly. Curing of epoxies to generate full cross-linking is a well-documented phenomenon [7]. In addition, subjecting the material to a thermal environment where the thermal performance of the composite increases would also increase the mechanical performance of the composite.

## 5. Conclusions

In this study we have demonstrated a simple method for determining damage in graphite-epoxy composites. The damage would be in the form of matrix cracks, fiber disbonds, or delaminations and would manifest itself in an apparent decrease in thermal conductivity. The equipment and procedure allow use of the method in the field, provided that a sample of undamaged material was available for use as a baseline for the measurement. The measurements showed that for unidirectional composites, additional information about damage can be obtained by measuring at a point offset from the laser spot, both along and perpendicular to the fiber direction.
